# Supplementary material for: Synthesis and Anticancer Activity of 1,3,4-Thiadiazoles with 3-Methoxyphenyl Substituent
Source: Molecules. 2022 Oct 17;27(20):6977. doi: 10.3390/molecules27206977 (PMC9607157; doi:10.3390/molecules27206977)
Supplement: Supplementary file 1 [file molecules-27-06977-s001.zip › molecules-1930283-supplementary.pdf]

Supplementary Information

Synthesis and anticancer activity of 1,3,4-thiadiazole with 3-methoxyphenyl substituent.

Sara Janowska[1], Dmytro Khylyuk[1], and Monika Wujec[1,*]

[1]Department of Organic Chemistry, Faculty of Pharmacy, Medical University,
4a Chodzki Str., 20-093 Lublin, Poland;

Table of contents:

1. ^{1}H NMR spectra of thiosemicarbazide derivatives (SC1-SC6)……………………………..……………………………………2
2. ^{1}H NMR spectra of 1,3,4-thiadiazole derivatives (STC1-STC6)……………………………………………………………………….8
3. ^{13}C NMR spectra of thiosemicarbazide derivatives (SC1-SC6)……….……………………………………………………………...13
4. ^{13}C NMR spectra of 1,3,4-thiadiazole derivatives (STC1-STC6)……………..…………………………………………………..19

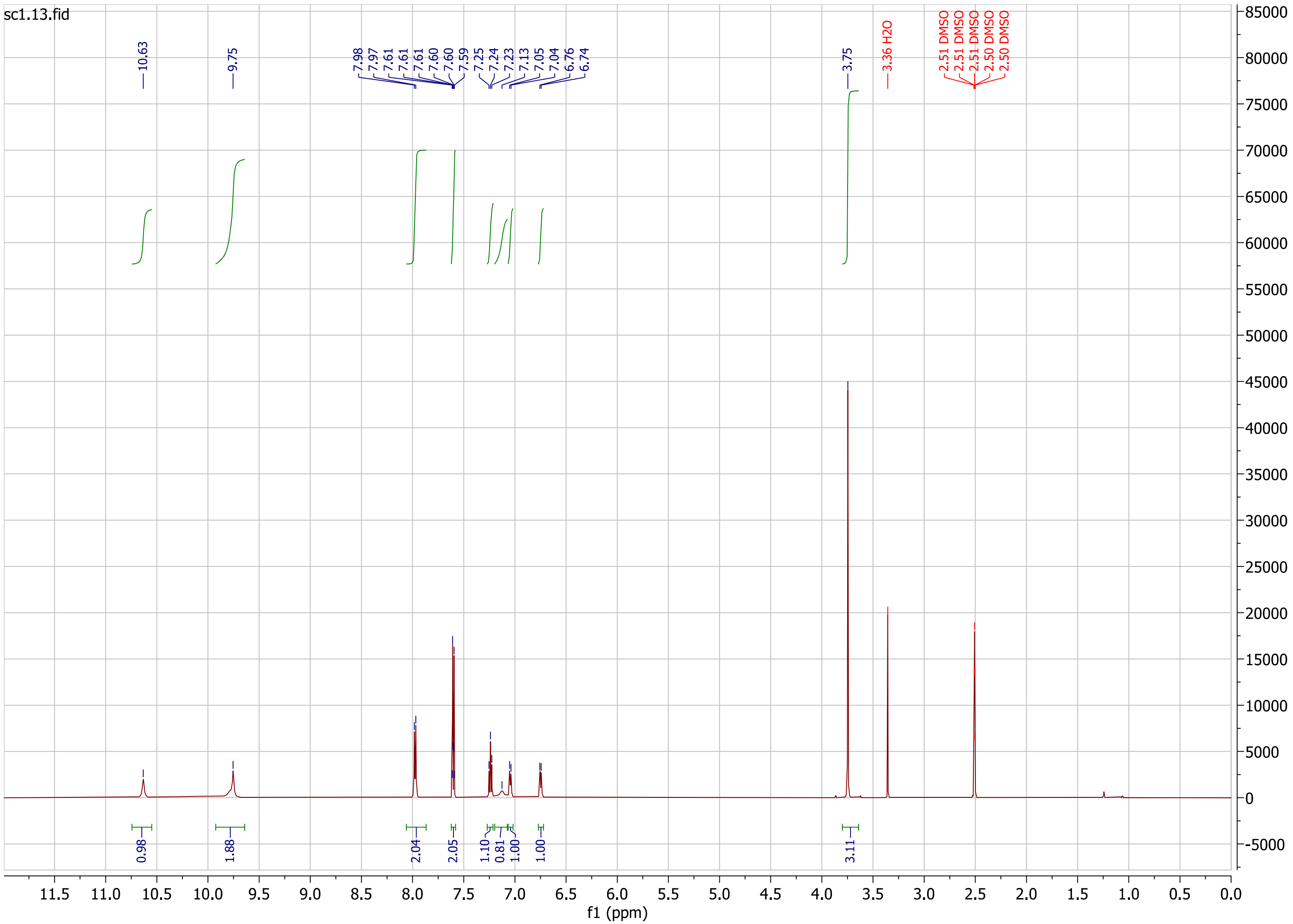

Figure S1. The ^{1}H NMR of compound SC1.

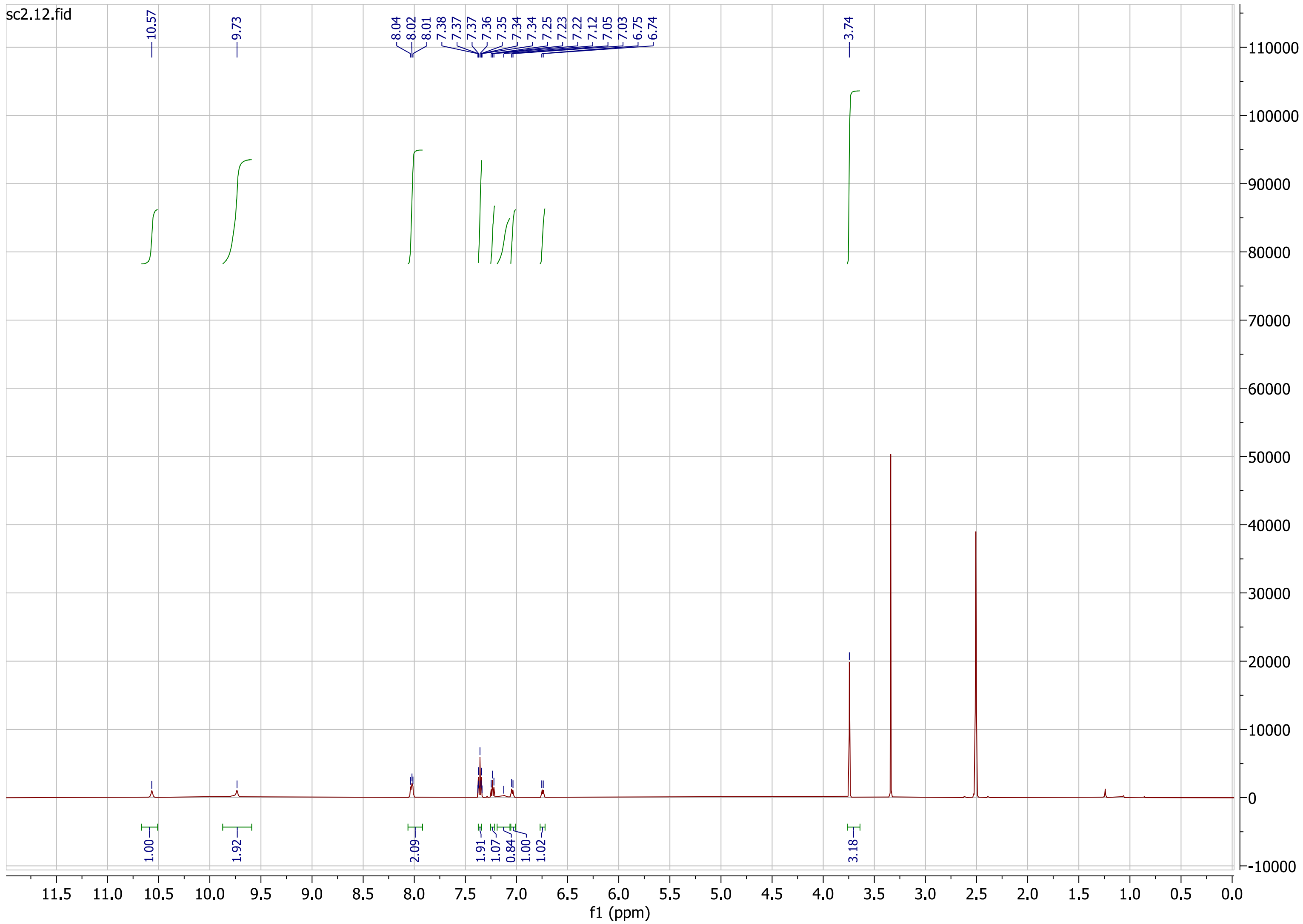

Figure S2. The ^{1}H NMR of compound SC2.

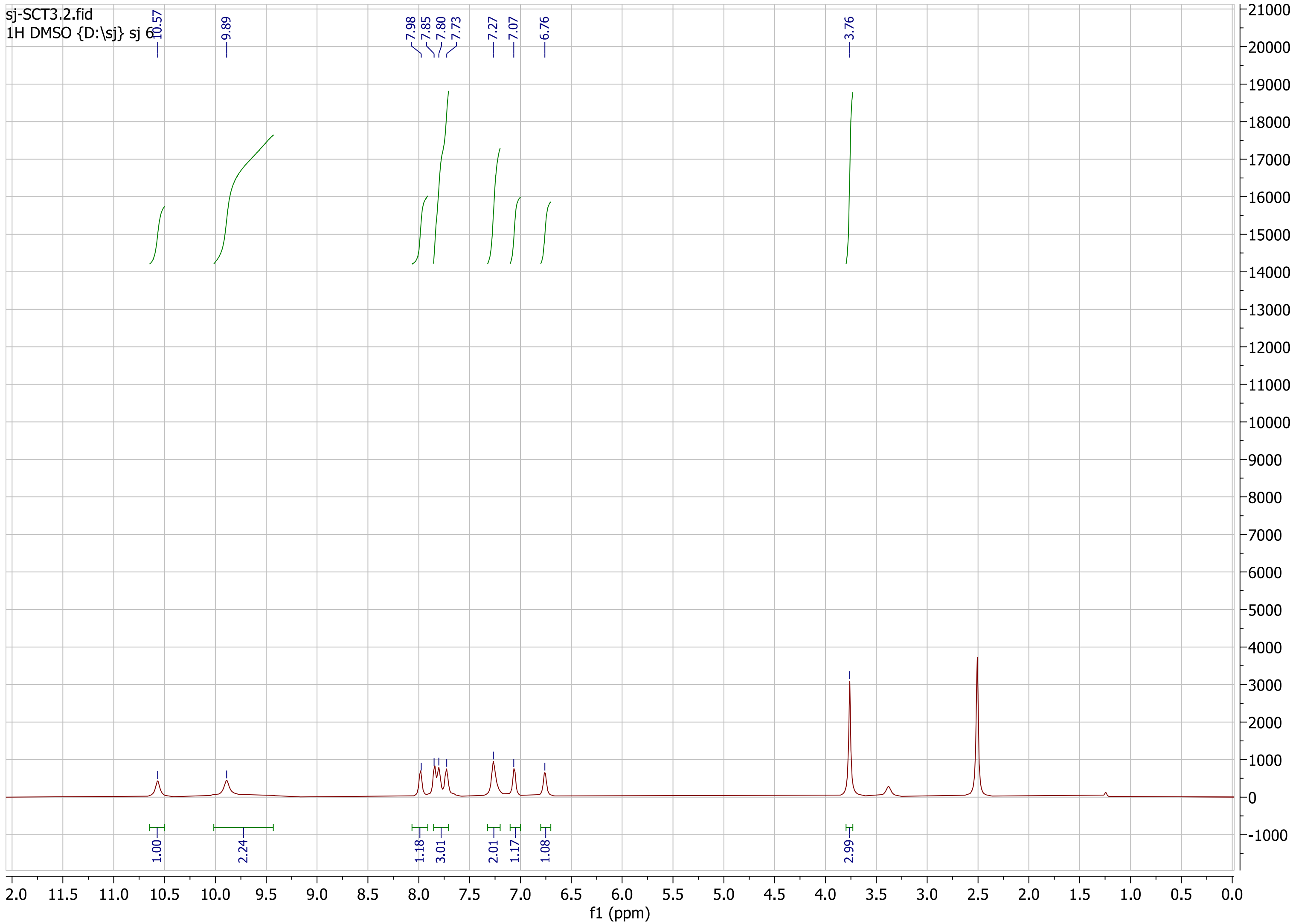

Figure S3. The ¹H NMR of compound SC3.

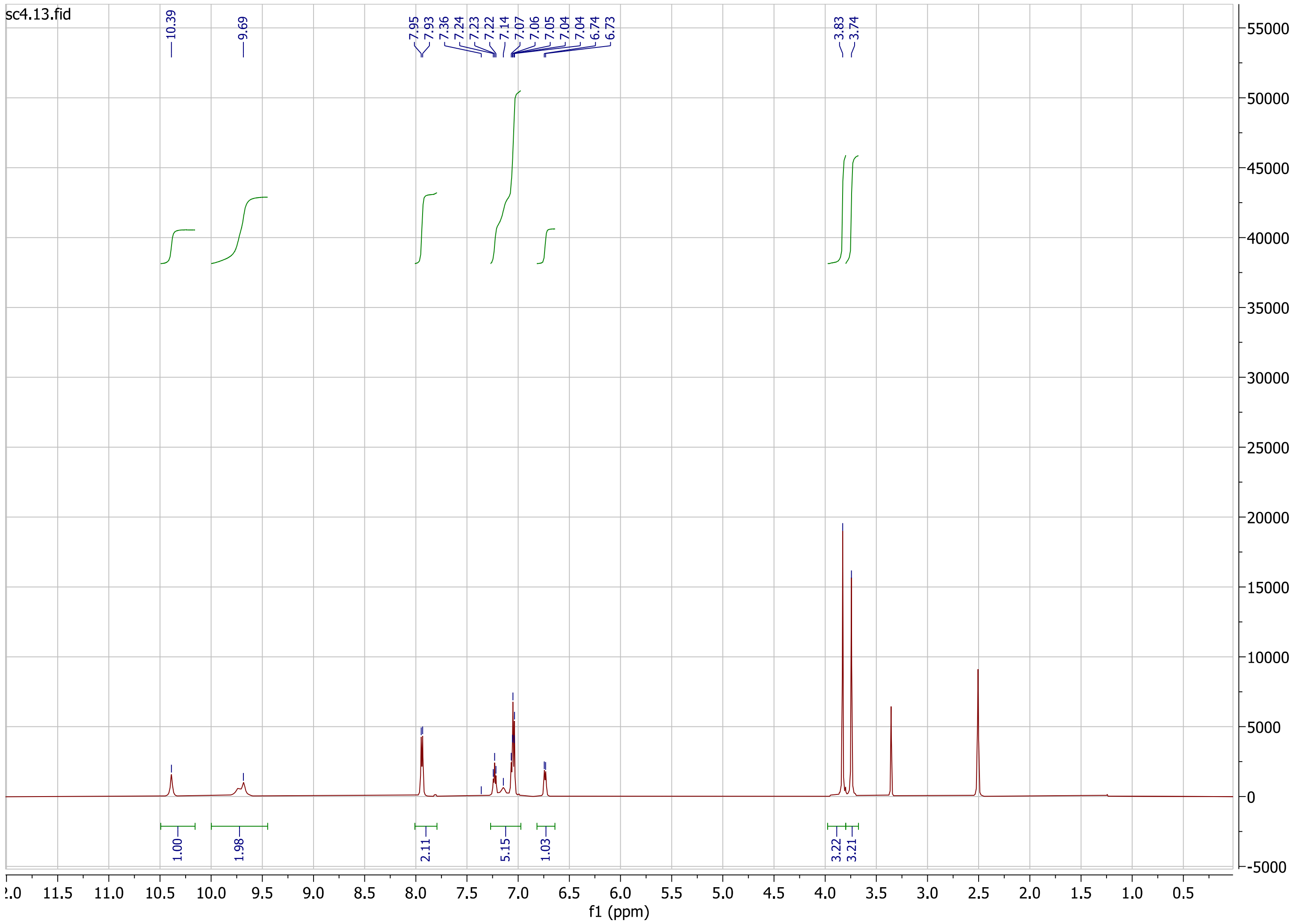

Figure S4. The ^{1}H NMR of compound SC4.

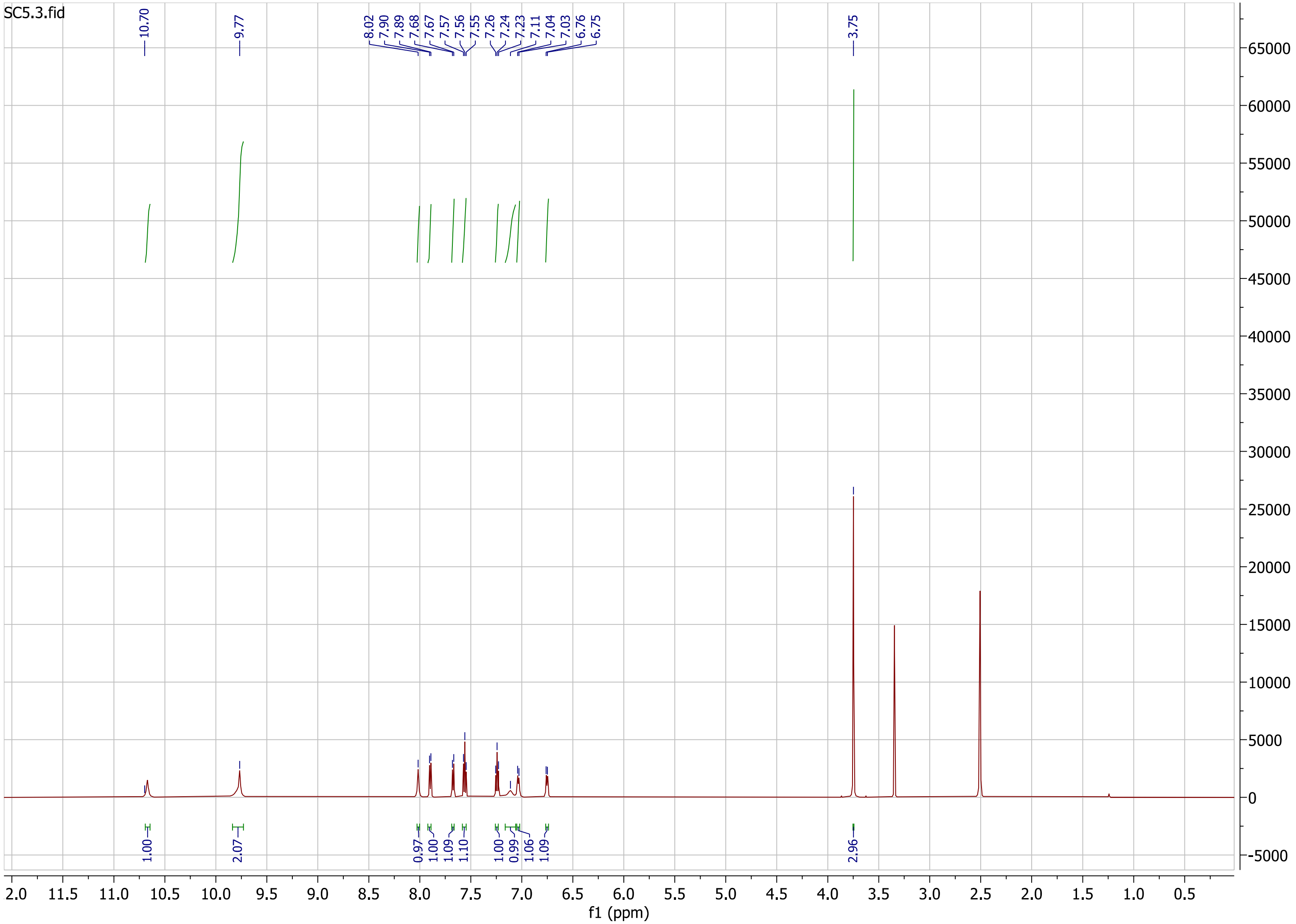

Figure S5. The ^{1}H NMR of compound SC5.

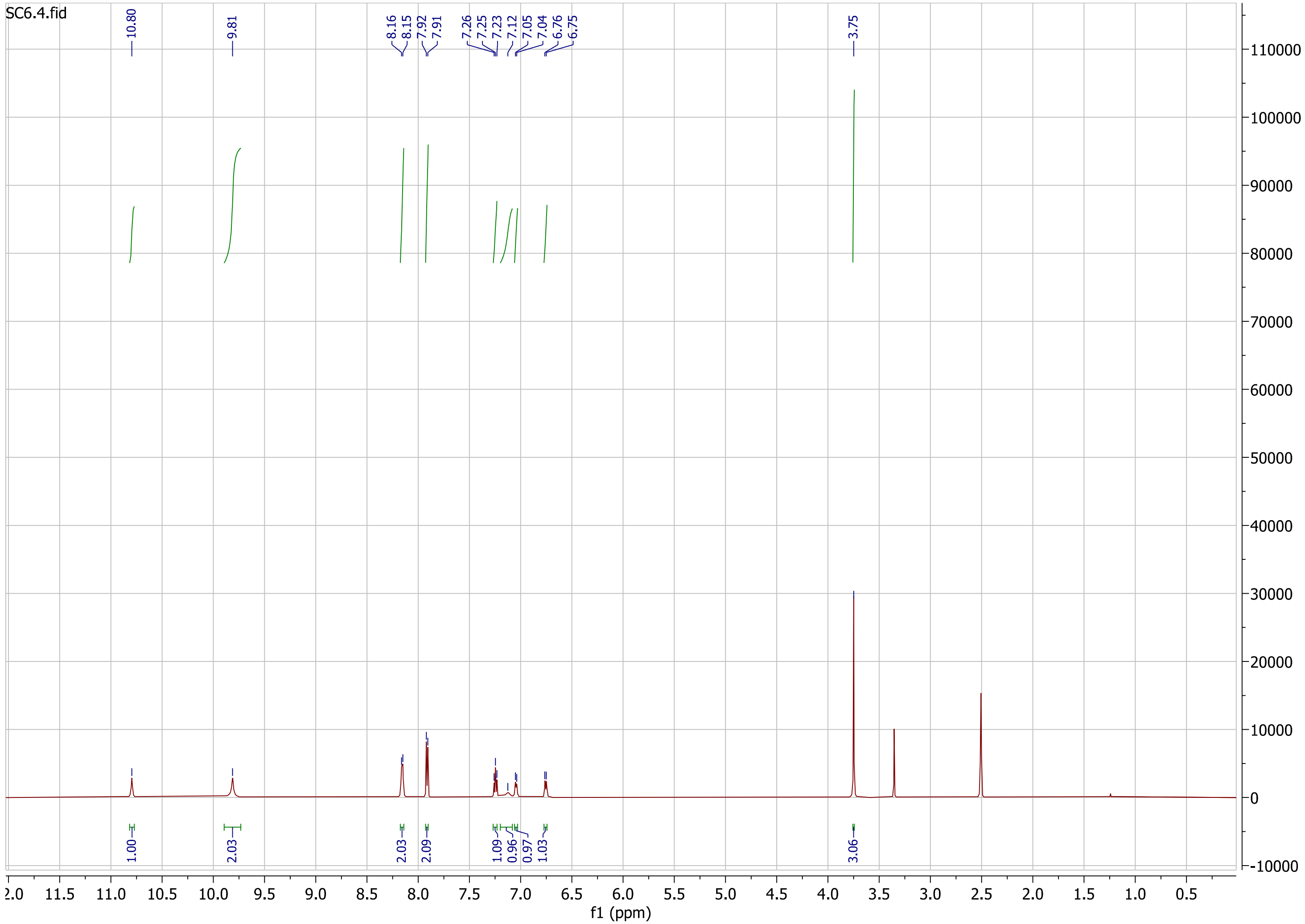

Figure S6. The ^{1}H NMR of compound SC6.

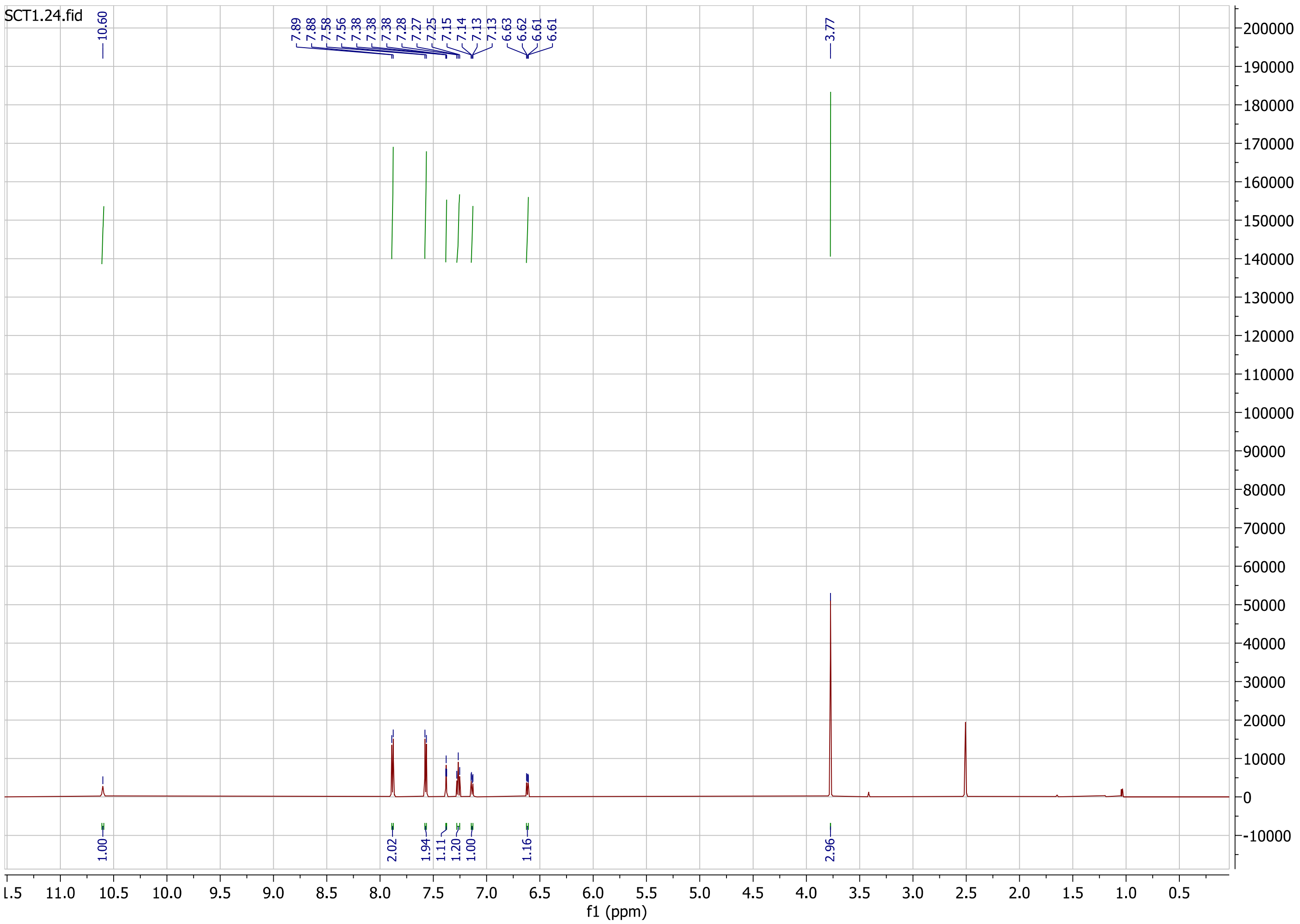

Figure S7. The ^{1}H NMR of compound SCT1.

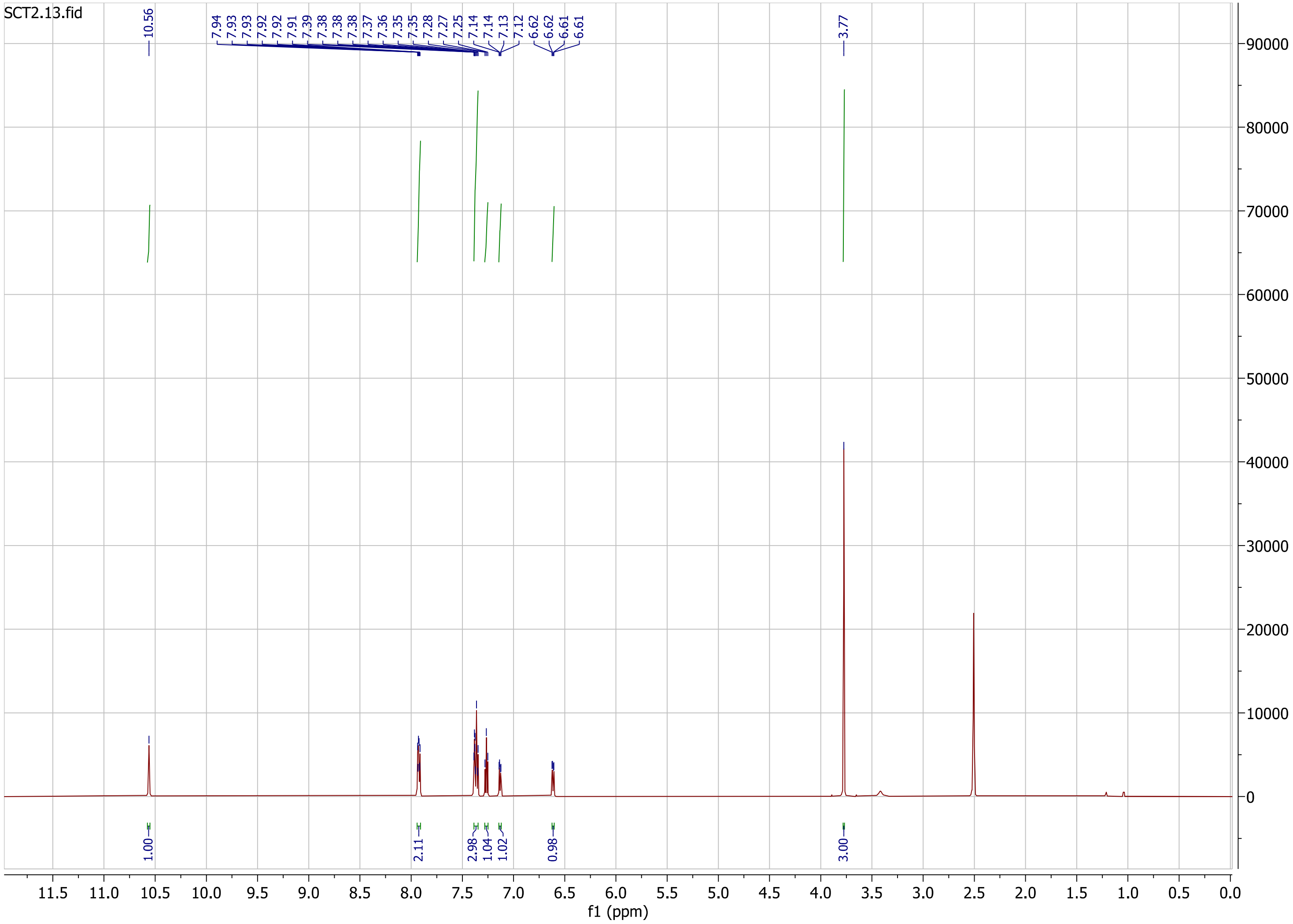

Figure S8. The ¹H NMR of compound SCT2.

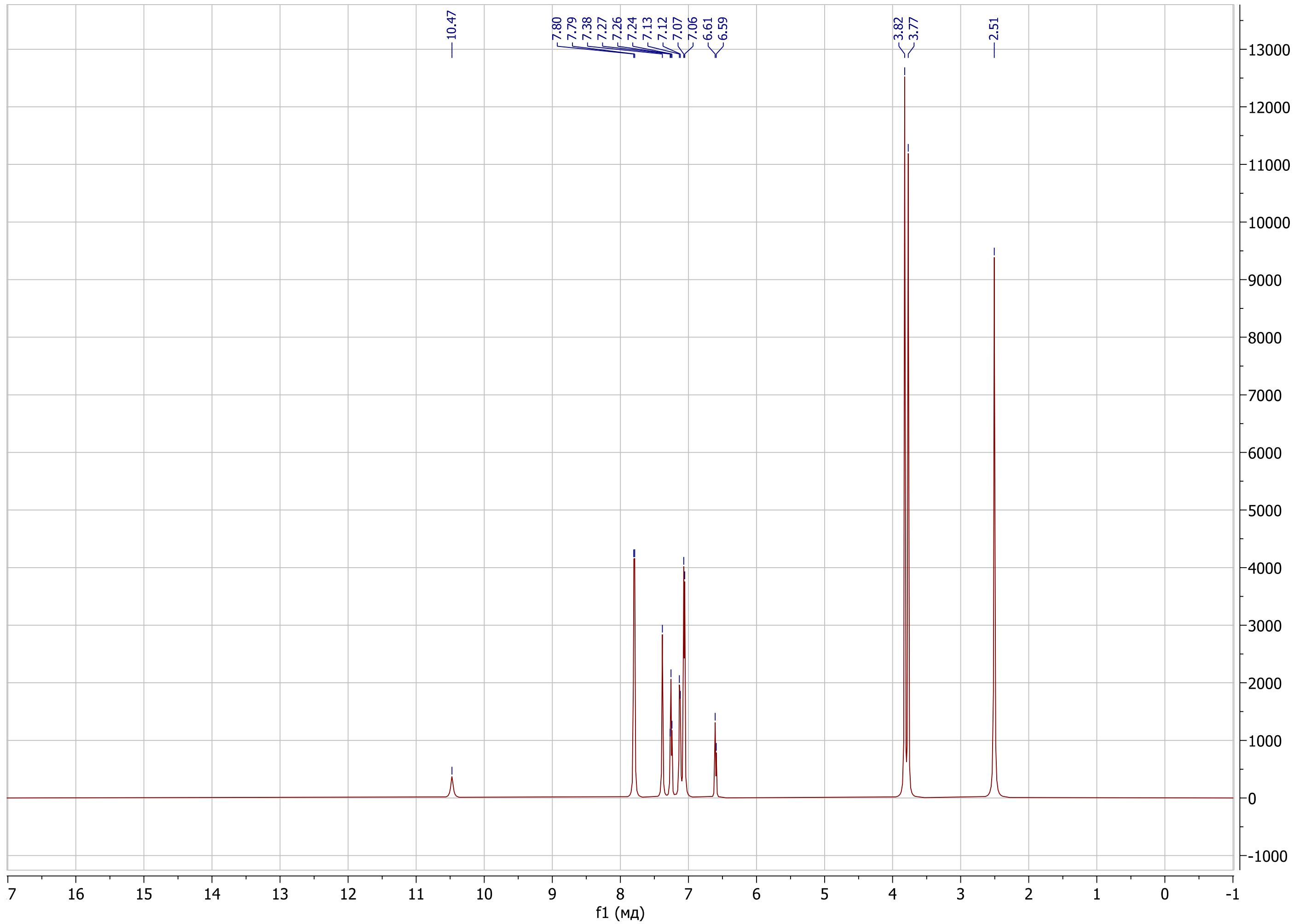

Figure S9. The ^{1}H NMR of compound SCT4.

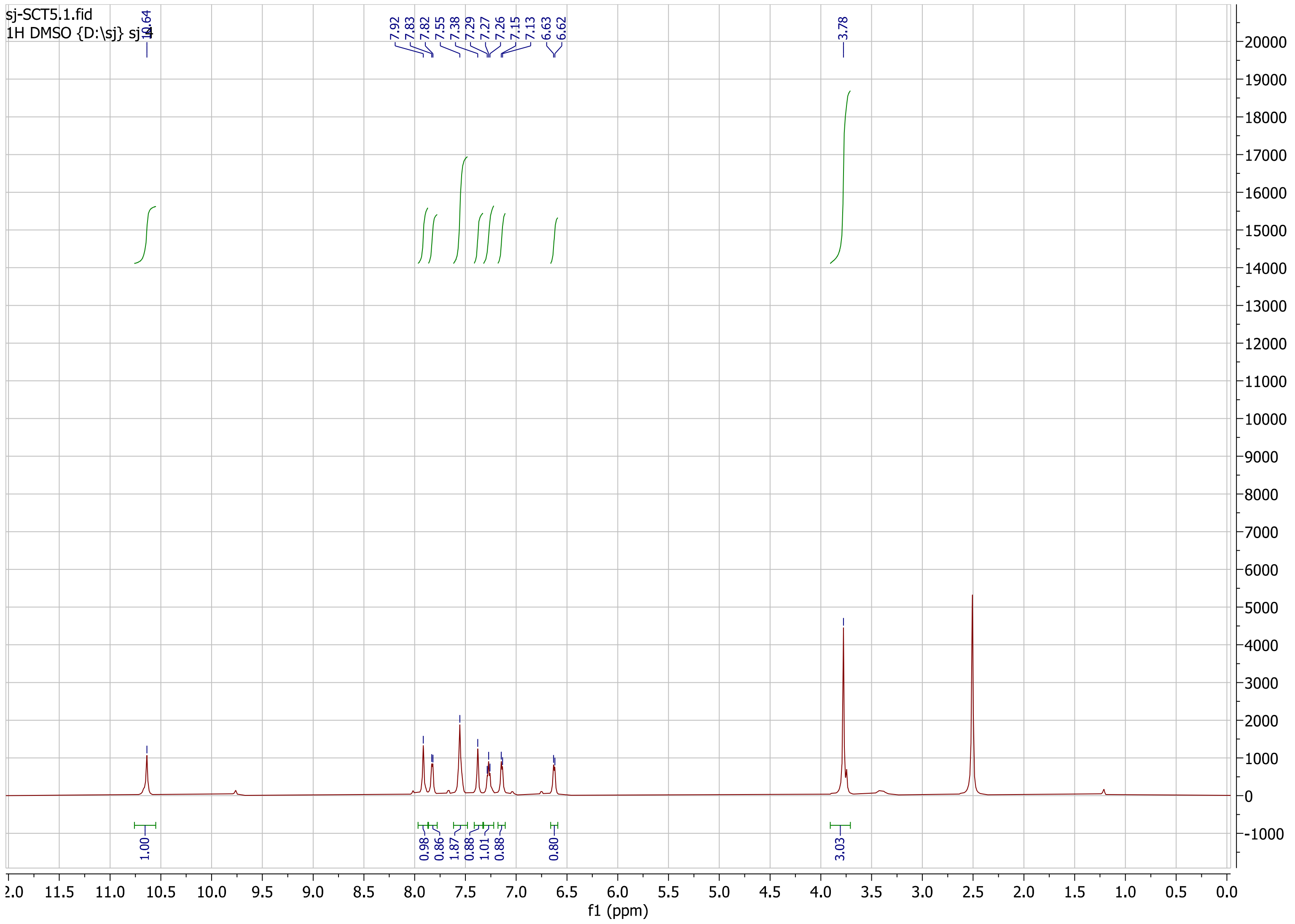

Figure S10. The ^{1}H NMR of compound SCT5.

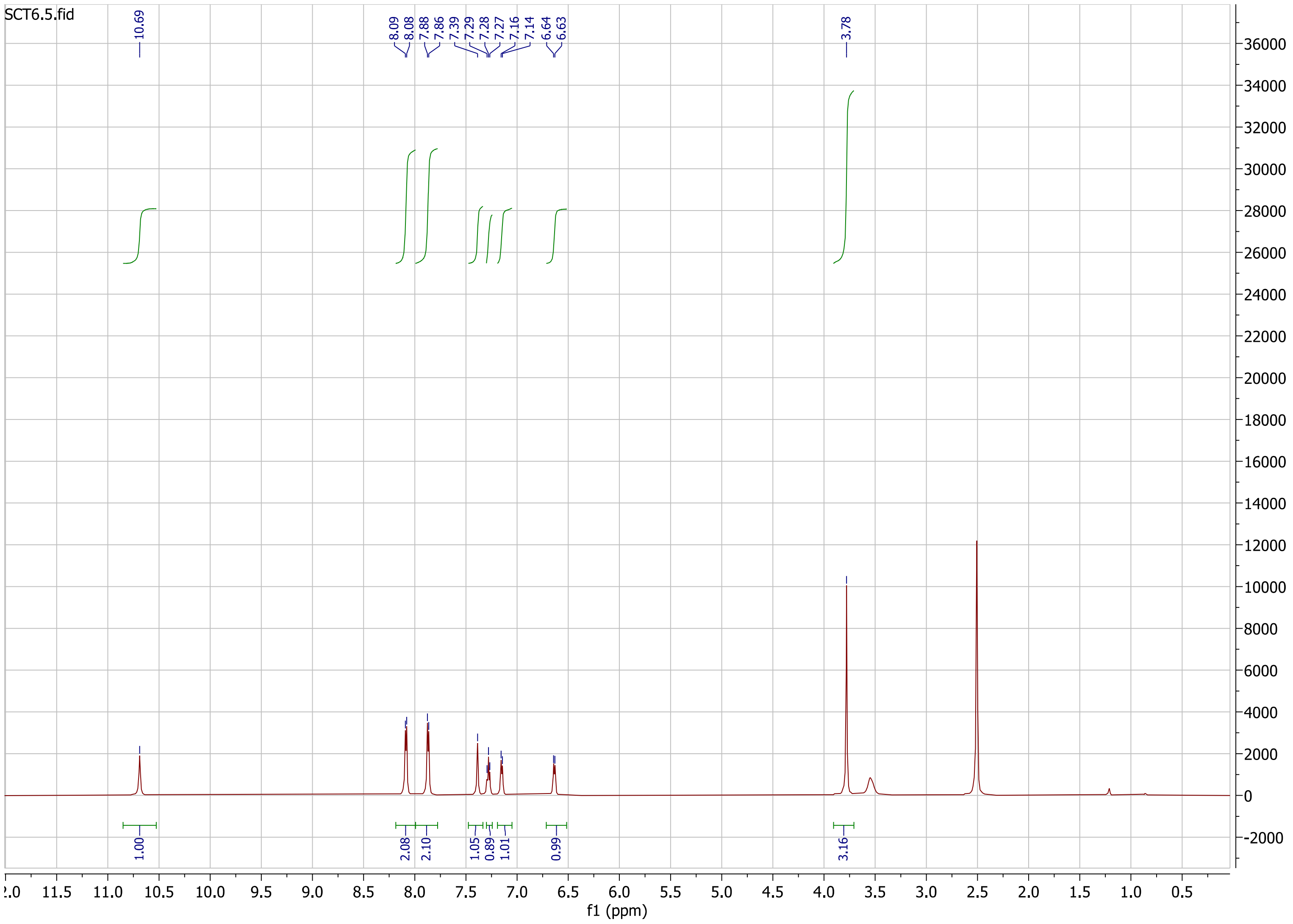

Figure S11. The ^{1}H NMR of compound SCT6.

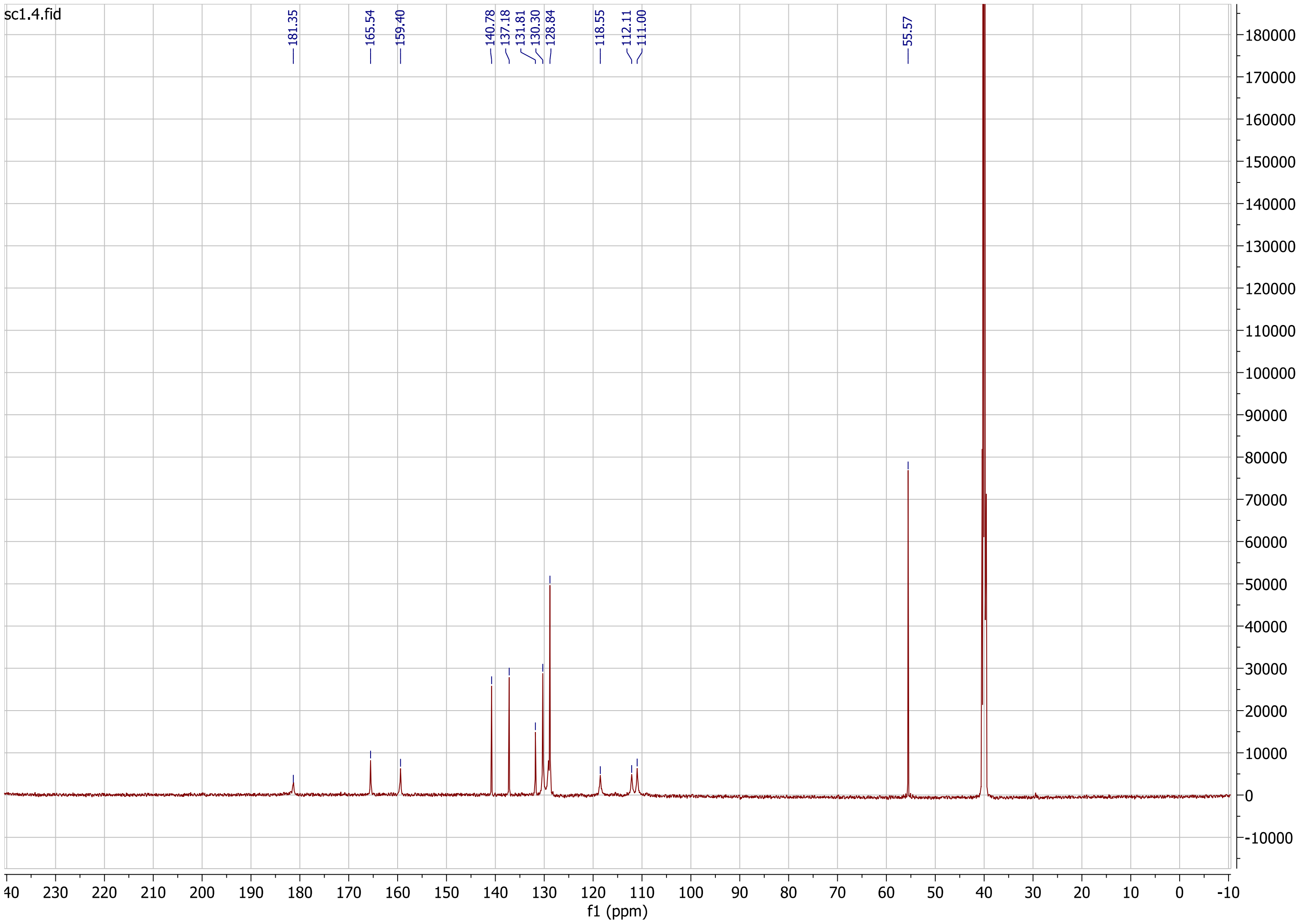

Figure S12. The ^{13}C NMR of compound SC1.

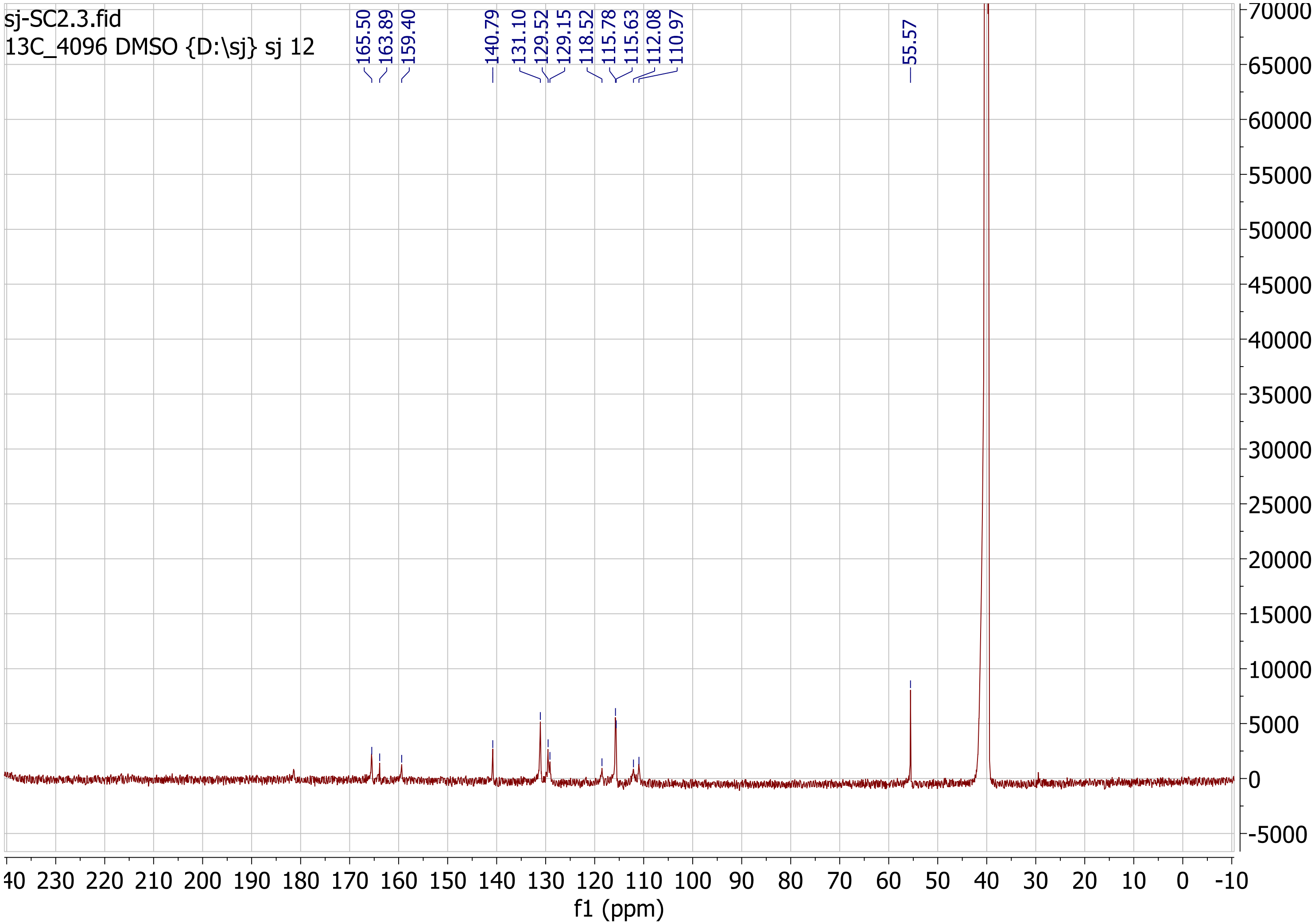

Figure S13. The ^{13}C NMR of compound SC2.

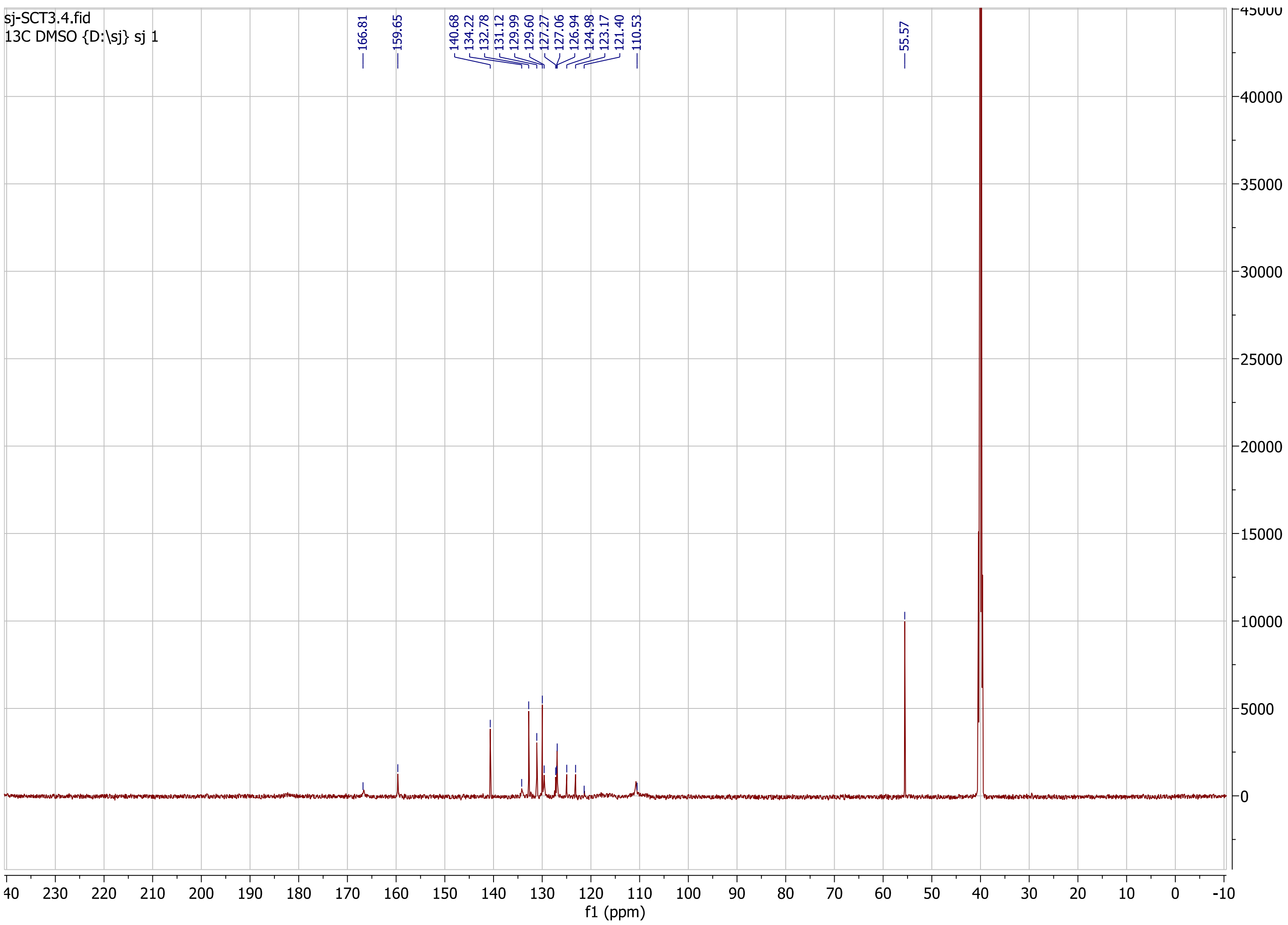

Figure S14. The ^{13}C NMR of compound SC3.

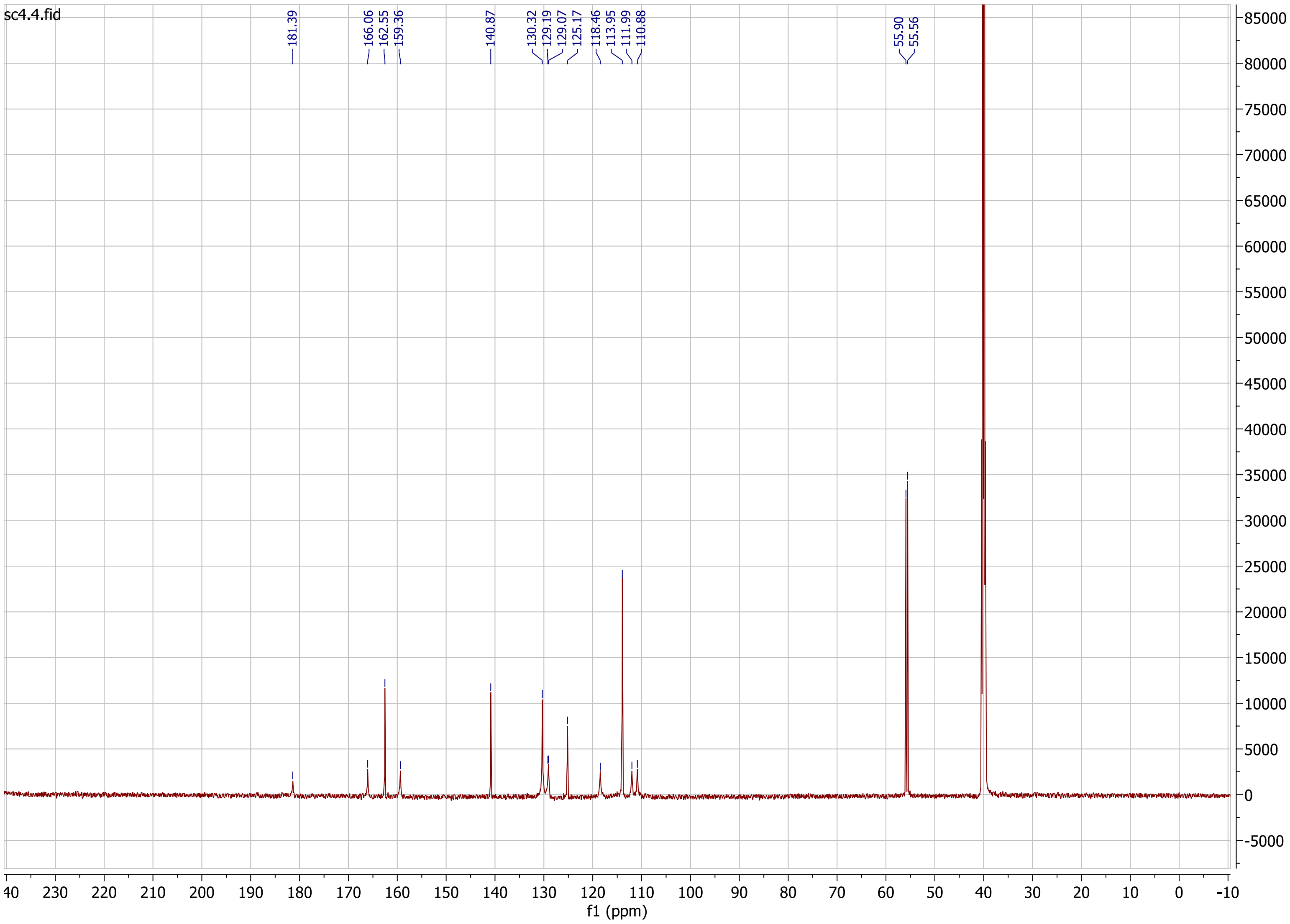

Figure S15. The ^{13}C NMR of compound SC4.

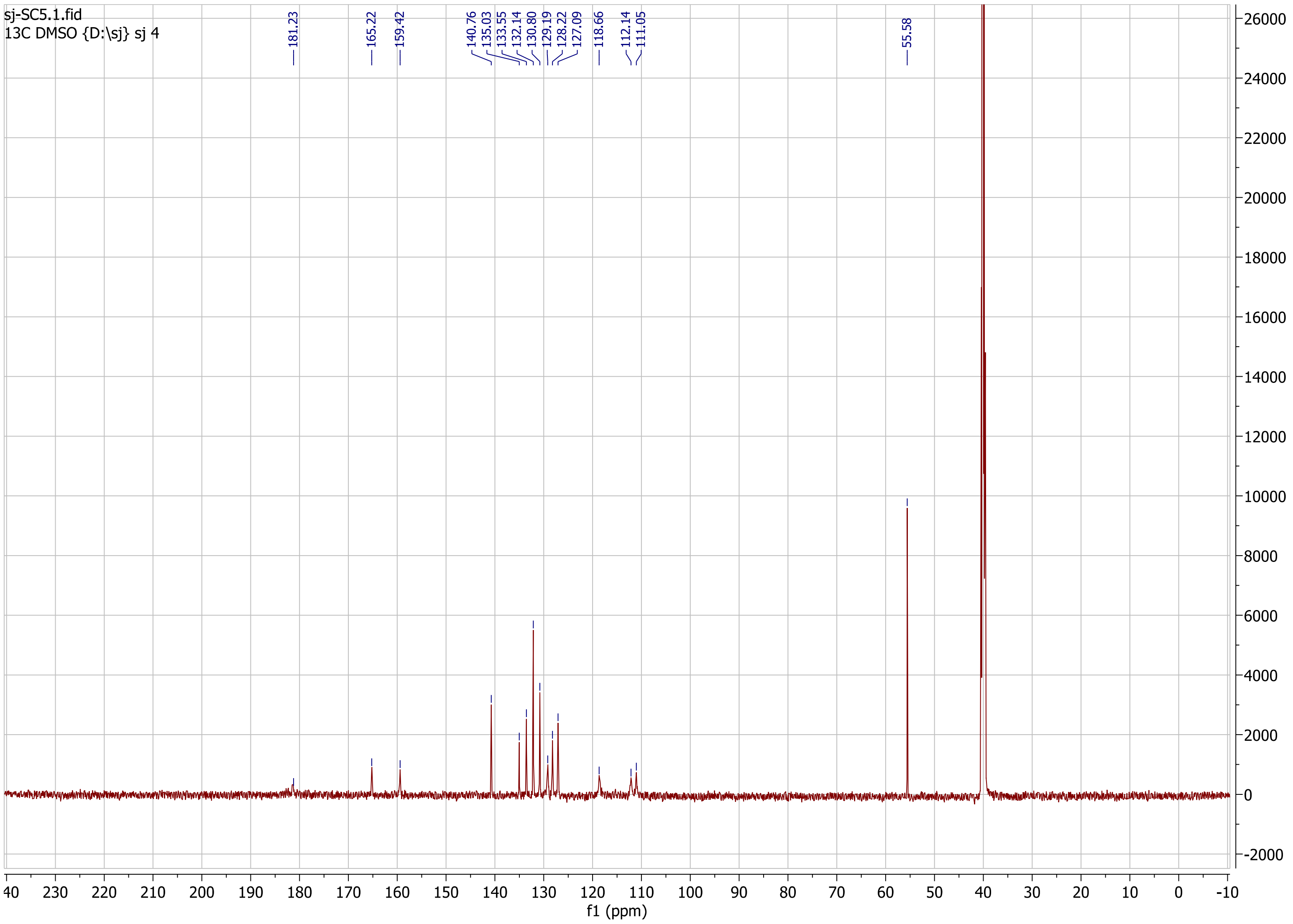

Figure S16. The ^{13}C NMR of compound SC5.

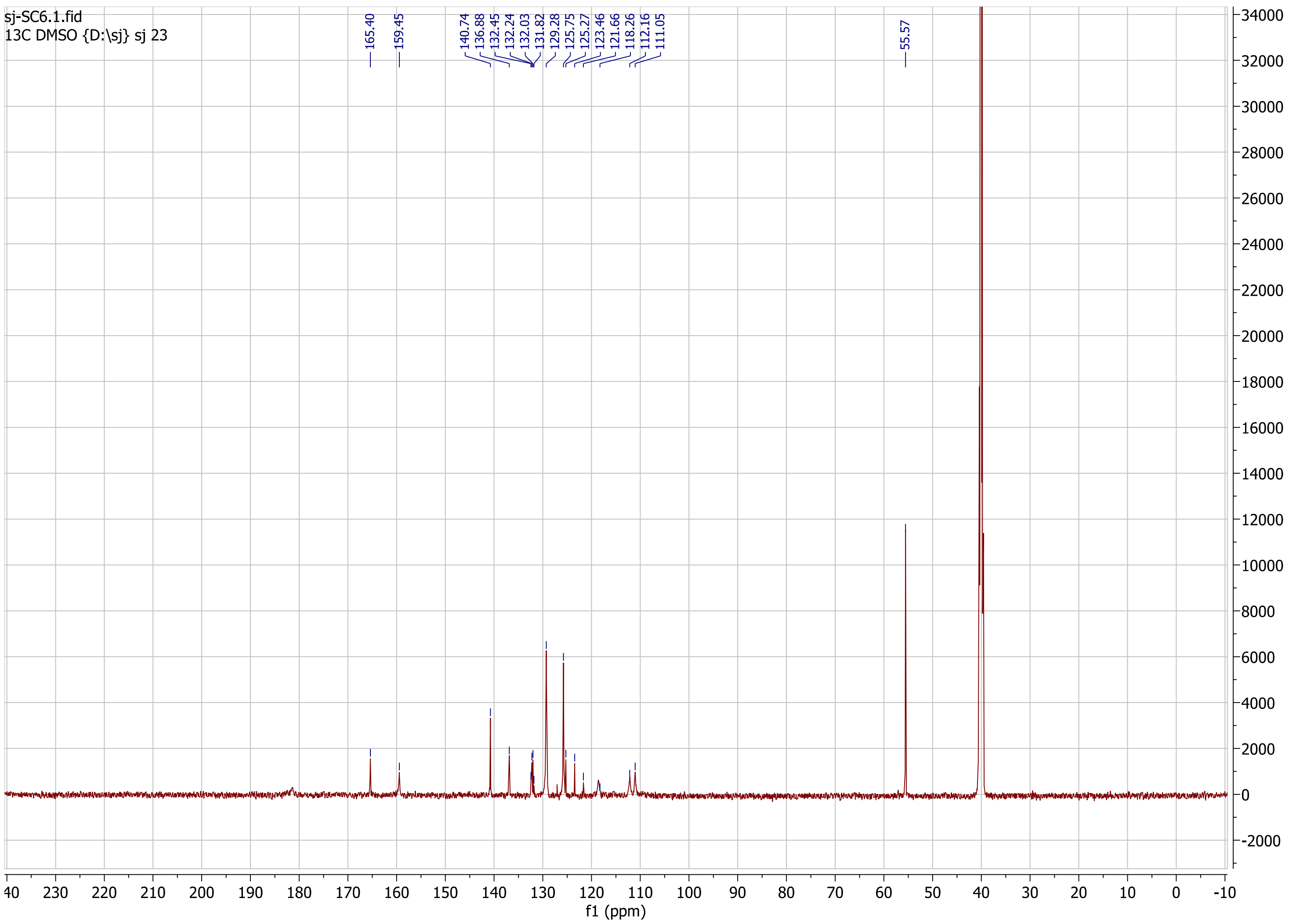

Figure S17. The ^{13}C NMR of compound SC6.

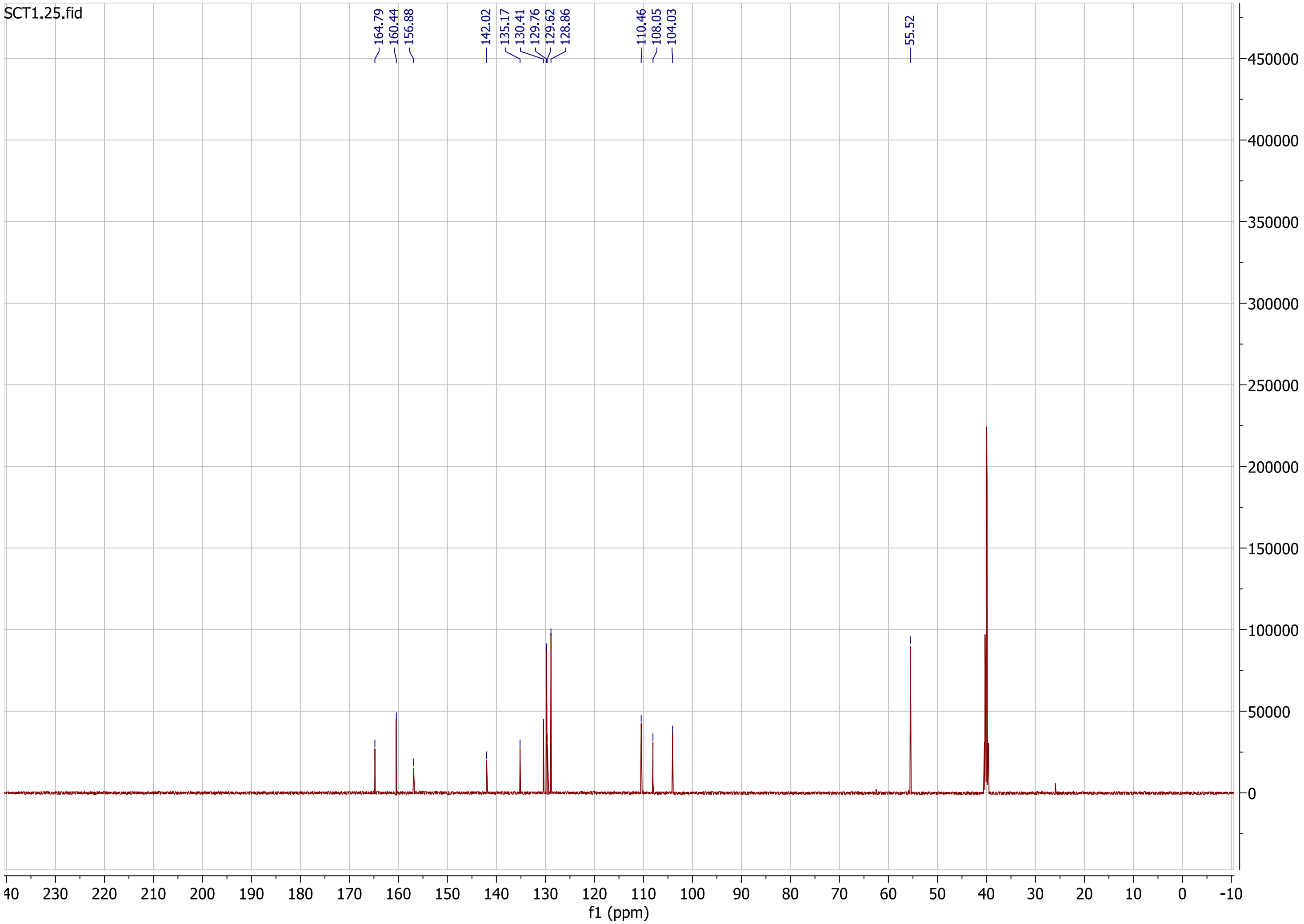

Figure S18. The ^{13}C NMR of compound SCT1.

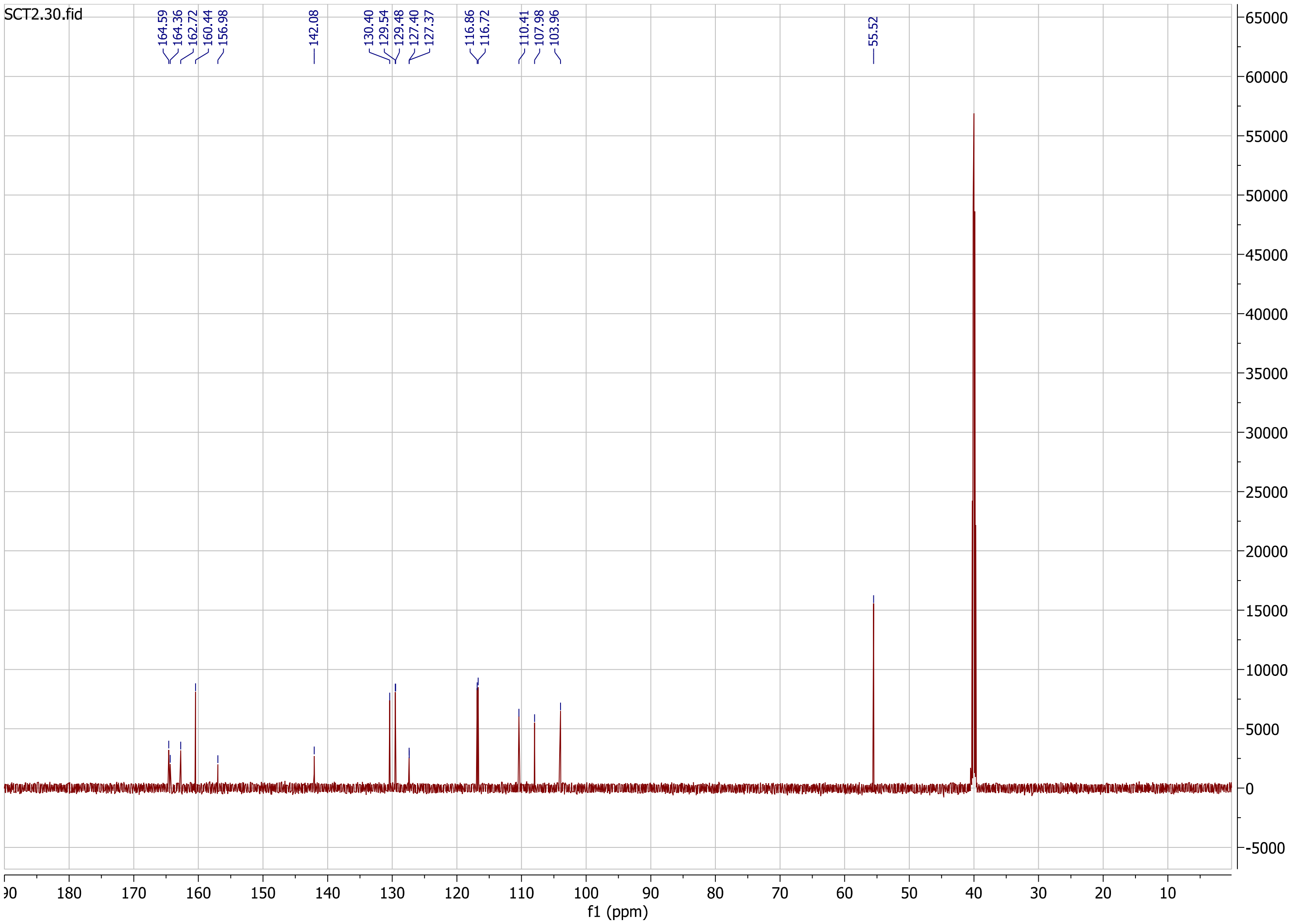

Figure S19. The ^{13}C NMR of compound SCT2.

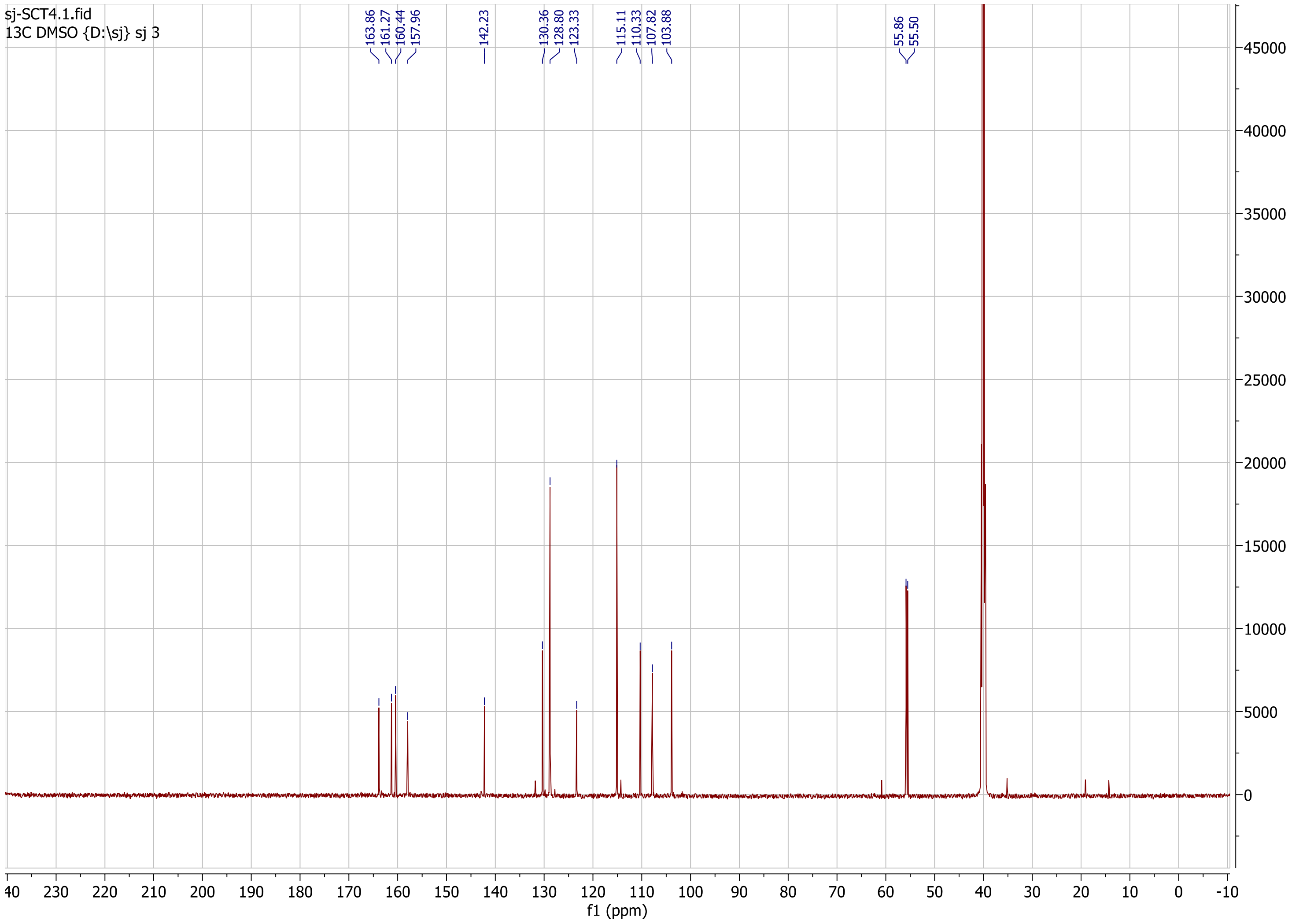

Figure S20. The ^{13}C NMR of compound SCT4.

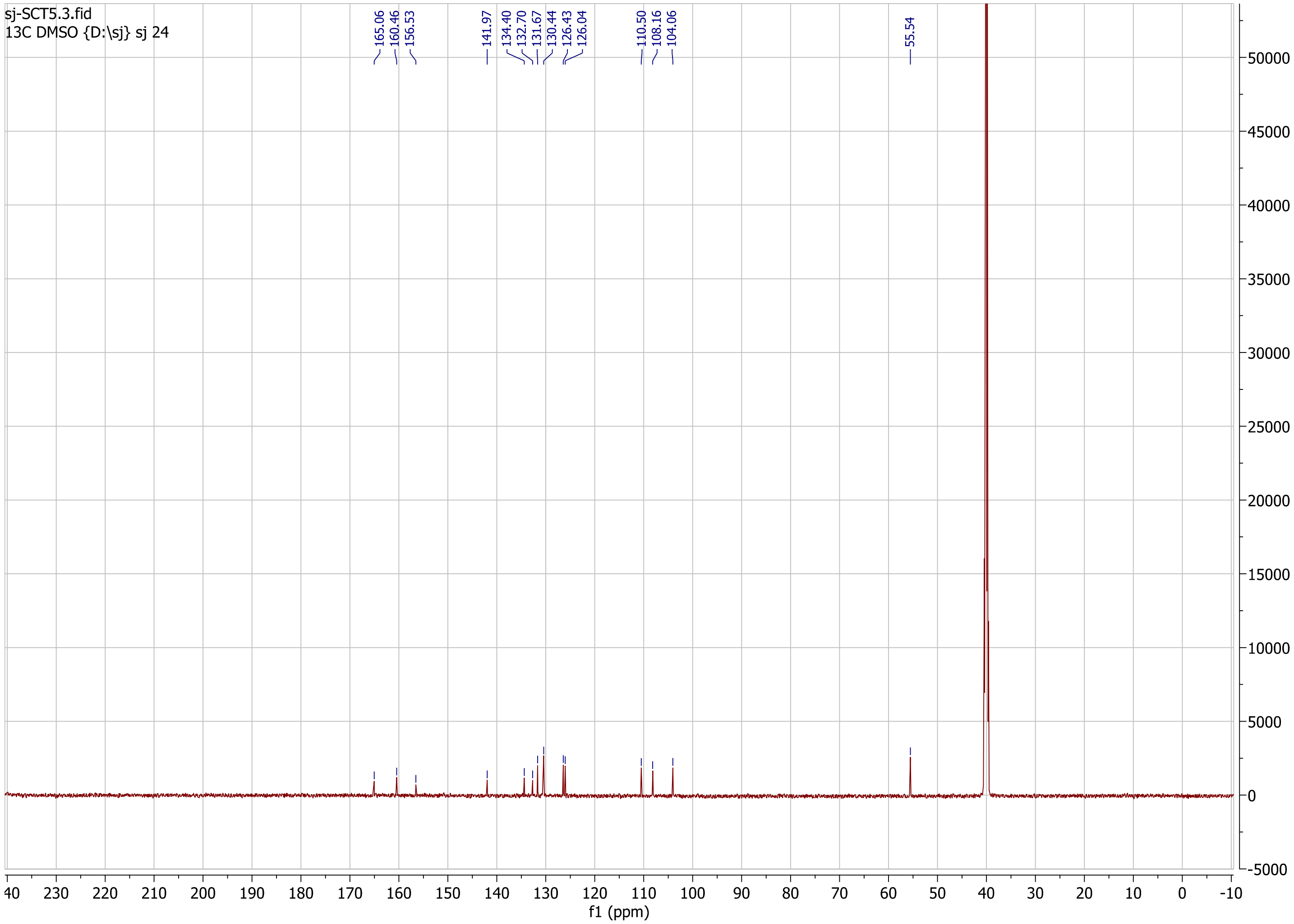

Figure S21. The ^{13}C NMR of compound SCT5.

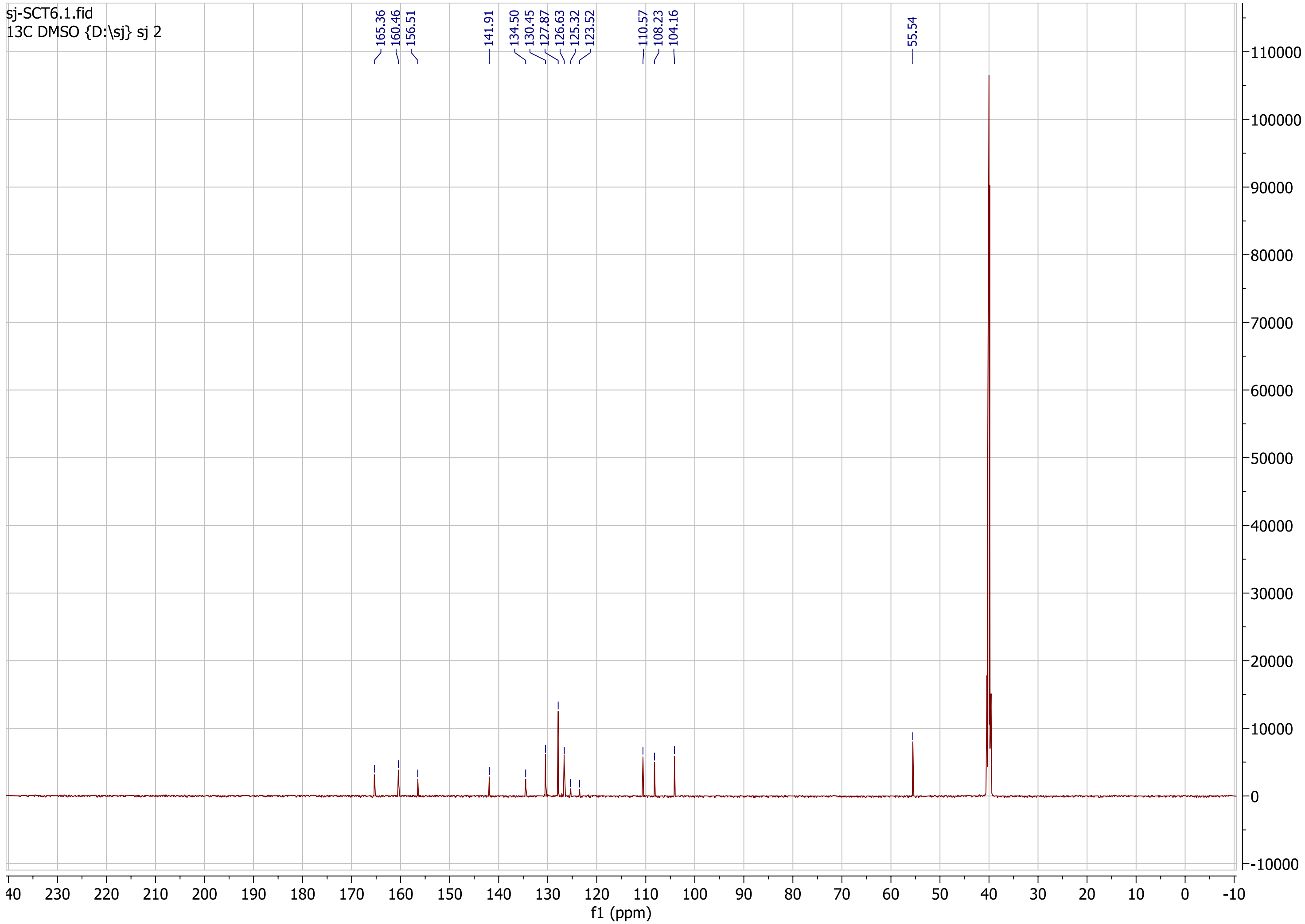

Figure S22. The ^{13}C NMR of compound SCT6.
